# A Novel Fluorescent Sensor for Fe^3+^ Based on a Quinoline Derivative

**DOI:** 10.3390/molecules30071579

**Published:** 2025-04-01

**Authors:** Xinru Zhang, Bo Sun, Huan Zhang, Chen Zhou, Qingqing Pan, Yining Wang, Chenyang Zou, Juan Hou, Jing Sun

**Affiliations:** 1 School of Chemistry & Environmental Engineering, Jilin Provincial International Joint Research Center of Photo-Functional Materials and Chemistry, Changchun University of Science and Technology, Changchun 130022, China; 2 China National Petroleum Corporation Hohhot Petrochemical Branch, Hohhot 010010, China

**Keywords:** ferric ion, fluorescent sensor, theoretical calculation

## Abstract

A new fluorescent sensor for detecting Fe^3+^ was developed based on chemical modification of the quinoline group. Titration experiments showed that the sensor exhibits high selectivity and sensitivity toward Fe^3+^, even in complex systems. The recognition mechanism was verified through theoretical calculations, demonstrating that the sensor can perform qualitative and quantitative analysis on Fe^3+^. The cell imaging and zebrafish imaging experiments further prove the potential application of the sensor in the field of bioluminescence imaging.

## 1. Introduction

Fe^3+^ is an oxidized state of the iron element, which has various important effects on the human body, mainly in the following aspects: (i) Participation in oxygen transport: Fe^3+^ is an important component of hemoglobin, which is responsible for transporting oxygen from the lungs to various parts of the body; (ii) Participation in cellular respiration: Fe^3+^ participates in intracellular respiration and helps cells produce energy; (iii) Participation in DNA synthesis: Fe^3+^ plays a role in DNA synthesis and is crucial for cell growth and reproduction; (iv) Maintaining normal hematopoietic function: Fe^3+^ is one of the main raw materials for producing red blood cells and is essential for the hematopoietic process; (v) Enhancing immune function: Fe^3+^ is able to maintain normal levels of T lymphocytes, serum complement activity, phagocytic function and neutrophil bactericidal ability in the human body [1,2,3,4]. However, when the total content of Fe^3+^ in the body is too high, high concentrations of free Fe^3+^ can cause oxidative stress, leading to symptoms such as weakened immunity, gastrointestinal discomfort and iron poisoning [5,6,7]. Therefore, the intake and metabolism of iron needs to be maintained within an appropriate range (10–15 mg daily) to maintain human health. Therefore, it is particularly important to develop methods with excellent detection capabilities for Fe^3+^. The common methods for detecting Fe^3+^ include atomic absorption spectroscopy, inductively coupled plasma mass spectrometry, flow injection analysis, voltammetry and microbiology [8,9,10]. However, the above methods often have disadvantages, such as complex operating procedures, high detection costs, limited sensitivity and long analysis time, which greatly limit their practical applications [11,12,13,14]. With the development of fluorescence detection technology, fluorescence sensors, as a new type of Fe^3+^ detection method, have shown advantages, such as high sensitivity, strong selectivity, fast response speed, low cost, real-time analysis and good portability, which is capable of effectively replacing the traditional detection methods mentioned above [15,16,17,18].

Quinoline is an aromatic organic compound with weak basicity, which is able to react with electrophilic and nucleophilic reagents to derive various compounds. Quinoline derivatives are capable of binding with metal ions to form complexes, thereby altering their fluorescence properties. Due to its rigid structure and co-ordination of fluorescent groups, quinoline and its derivatives are widely used for the detection of metal ions [19]. At the same time, quinoline derivatives are also used for fluorescence imaging of cells and tissues due to their good water solubility and biocompatibility [20]. For example, certain quinoline nitrile derivatives exhibit infrared to near-infrared (NIR) emission characteristics, which make them highly signal-to-noise ratio and penetrative derivatives in biological imaging [21]. Therefore, quinoline fluorophores, as an important fluorescent marker, have broad application prospects in fields such as biomedical and materials science. By designing and synthesizing novel quinoline compounds, researchers are able to develop more sensitive and specific fluorescent probes for substance detection and biological research. In order to improve the selectivity of quinoline derivatives and the co-ordination ability with metal ions, we introduced N-containing groups through chemical reactions. In addition, in order to prevent unnecessary reactions of amino groups in the recognition process, so as to ensure the smooth detection of metal ions, trifluoroacetyl was used to protect amino groups.

In this study, a new fluorescent sensor 2,2,2-trifluoro-*N*′-(2-(quinolin-8-yloxy) acetyl)acetohydrazide (TQA) was successfully designed and synthesized via chemical modification of quinoline fluorescent groups and applied to the detection of Fe^3+^. The experimental results showed that Sensor TQA exhibited high selectivity and sensitivity toward Fe^3+^ and could effectively recognize Fe^3+^ in complex systems. Furthermore, the potential application of Sensor TQA in fluorescence imaging of living cells and zebrafish was further investigated.

## 2. Results

### 2.1. Spectral Properties and Research of Sensor TQA

**Figure 1:**
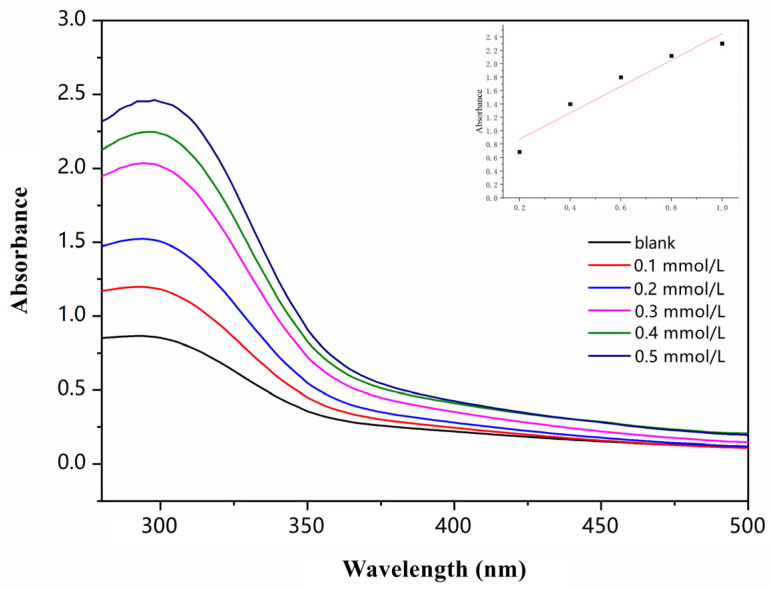
UV–visible spectra of Sensor TQA (0.5 mmol/L) in the presence of different concentrations of Fe^3+^ (0.1 mmol/L–0.5 mmol/L) in DMF/water solution (*v*:*v* = 1:1, pH = 7.4).

The UV–visible absorption spectrum of Sensor TQA for different concentrations of Fe^3+^ is illustrated in Figure 1. Sensor TQA was prepared in a 0.5 mmol/L DMF: water (1:1, *v*:*v*) solution. The maximum absorption peak of blank Sensor TQA appeared at 301 nm. Under the same testing environment, 0.1 mmol/L, 0.2 mmol/L, 0.3 mmol/L, 0.4 mmol/L, 0.5 mmol/L of Fe^3+^ were added to the solution of Sensor TQA. As the Fe^3+^ concentration increased, absorbance at 301 nm gradually increased, and the linear fitting of different concentrations and absorbance at 301 nm were carried out; therefore, in subsequent tests, 301 nm was selected as the excitation wavelength for fluorescence emission spectroscopy testing.

**Figure 2:**
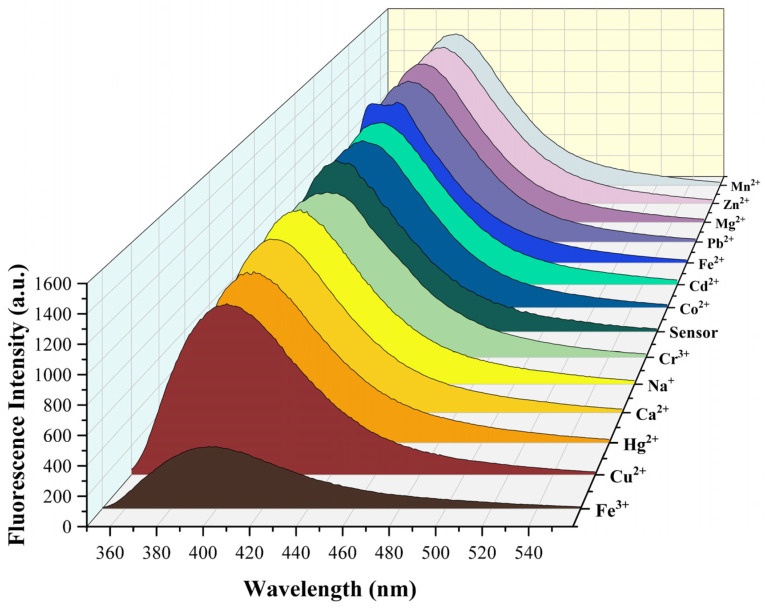
Fluorescence response of Sensor TQA (0.5 mmol/L) to different metal ions (0.5 mmol/L) in DMF/water solution (*v*:*v* = 1:1, pH = 7.4, λ_ex_ = 301 nm).

Next, in order to test the selectivity of Sensor TQA, the prepared metal ion solutions were dissolved in a HEPES-NaOH buffer solution (pH = 7.4). Sensor TQA solution was prepared in *N*,*N*-Dimethylformamide (DMF). The solvent condition in the experiment is that the ratio of organic phase to aqueous phase is 1:1. Solutions of common metal ions (Na^+^, Mg^2+^, Zn^2+^, Cd^2+^, Ca^2+^, Mn^2+^, Pb^2+^, Cu^2+^, Hg^2+^, Cr^3+^, Fe^3+^, Fe^2+^, Co^2+^, pH = 7.4) with equal volume and concentration were added to the solution of Sensor TQA (DMF: water = 5:5, *v*:*v*). The fluorescence responses of Sensor TQA (0.5 mmol/L) to different metal ions (0.5 mmol/L) are illustrated in Figure 2 (λ_ex_ = 301 nm). The maximum emission peak of blank Sensor TQA in the fluorescence spectrum appeared at 397 nm, and only Fe^3+^ significantly reduced the fluorescence intensity of Sensor TQA. However, the addition of other metal ions at the same concentration did not change the fluorescence intensity of Sensor TQA; therefore, it can be concluded that Sensor TQA has good selectivity for Fe^3+^. Subsequently, to further demonstrate the detection capability of Sensor TQA for Fe^3+^, tests were conducted in a more complex system. In addition, in order to verify whether common anions affect the fluorescence emission of Sensor TQA, common anions were added to the Sensor TQA solution under the same test conditions. As shown in Figure S7, the anions did not affect the fluorescence emission of the sensor.

**Figure 3:**
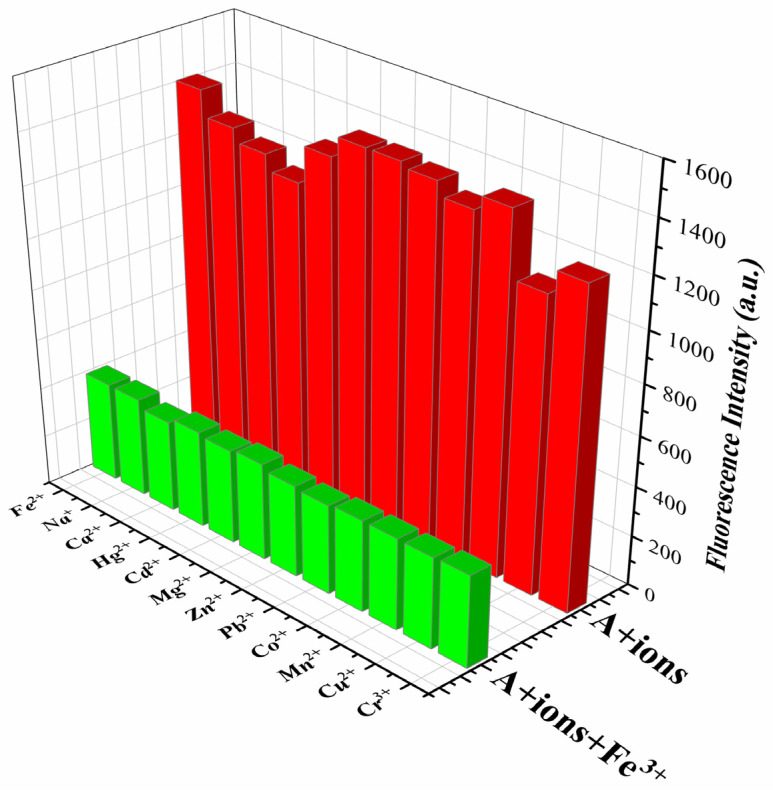
Competitive experiments (green bar) and selective experiments (red bar) on Sensor TQA for detecting Fe^3+^ in the presence of interfering metal ions in DMF/water solution (*v*:*v* = 1:1, pH = 7.4, λ_ex_ = 301 nm, λ_em_ = 397 nm).

The ability to effectively detect ions under complex conditions is an important criterion for evaluating the performance of fluorescence sensors. Therefore, a series of common metal cations were selected for competitive experiments on Sensor TQA. Before the experiment, the solution of Sensor TQA (0.5 mmol/L) was induced to undergo fluorescence quenching by equal volume and concentration of the Fe^3+^ solution (pH = 7.4). Then, 10 equivalents of other cations were added (Na^+^, Mg^2+^, Fe^2+^, Zn^2+^,Cd^2+^, Ca^2+^, Mn^2+^, Pb^2+^, Cu^2+^, Hg^2+^, Cr^3+^, Co^2+^). The emission spectrum was recorded with a fluorescence spectrophotometer. The maximum fluorescence intensity at 397 nm was selected as the data for the competitive experiment. The experimental results are shown in Figure 3. The green bar represents the competitive experimental data; in addition, the results of selective experiments (red bar) are also used as a reference. It can be seen that the introduction of 10 equivalents of other cations (5 mmol/L) did not significantly affect Fe^3+^ recognition based on fluorescence quenching. This indicates that Sensor TQA is able to produce a targeted fluorescence intensity response to Fe^3+^ under complex conditions, which also proves that Sensor TQA has good selectivity and anti-interference ability, and it provides a certain guarantee for detection under actual complex conditions. Therefore, Sensor TQA can be considered as a potentially valuable qualitative analysis tool for Fe^3+^.

**Figure 4:**
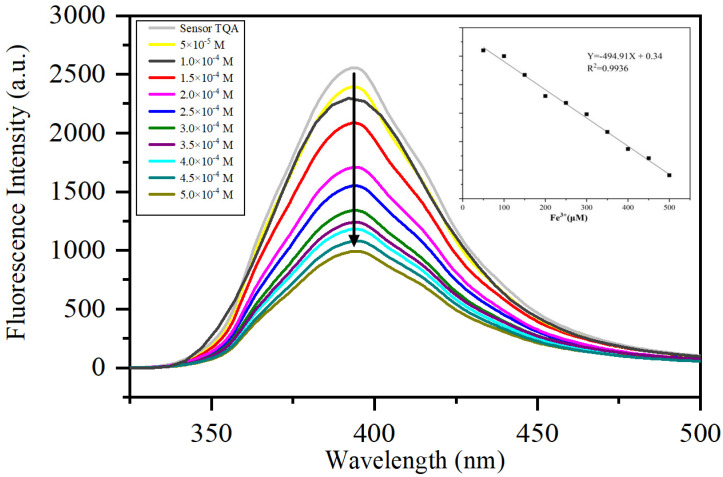
Fluorescence spectral response of Sensor TQA (0.5 mmol/L) to Fe^3+^ (0.05 mmol/L–0.5 mmol/L) at different concentrations in DMF/water solution (*v*:*v* = 1:1, pH = 7.4, λ_ex_ = 301 nm, λ_em_ = 397 nm).

In order to further determine the effect of Fe^3+^ on the fluorescence emission spectrum of Sensor TQA, the fluorescence spectrum response of fluorescent molecular Sensor TQA to Fe^3+^ for different concentration conditions (0.05 mmol/L~0.5 mmol/L) was studied. In Figure 4, it is easy to perceive that the fluorescence emission of the blank solution of Sensor TQA is very strong. As the concentration of Fe^3+^ added to the solution continues to increase, the fluorescence quenching effect of Sensor TQA becomes more and more obvious in the emission spectrum. When the Fe^3+^ concentration increases to 0.5 mmol/L, the fluorescence intensity of Sensor TQA is quenched by about 70%, indicating that Sensor TQA is completely bound to iron ions and has high sensitivity at this time. In order to determine whether Sensor TQA can carry out a quantitative analysis of Fe^3+^, the fluorescence intensity of Sensor TQA solution at 397 nm was selected and plotted against the corresponding Fe^3+^ concentration data. The fluorescence intensity of Sensor TQA solution showed a linear weakening trend with the increasing concentration of Fe^3+^, and a good linear relationship was observed. After linear fitting, the linear equation y = −494.91x + 0.34 (R^2^ = 0.9936) was obtained, indicating that Sensor TQA is able to perform quantitative detection of Fe^3+^.

**Figure 5:**
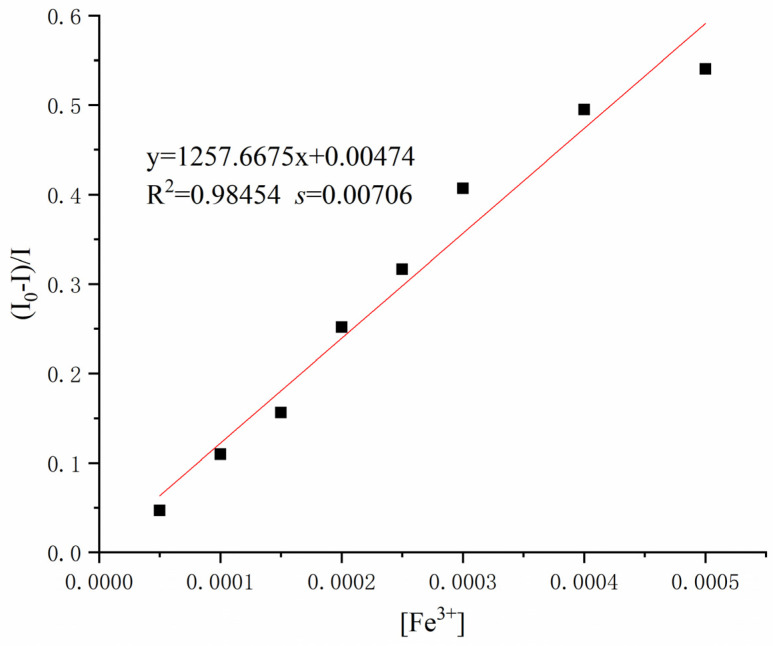
Linear fitting of the fluorescence titration curve of Sensor TQA in DMF/water solution (*v*:*v* = 1:1, pH = 7.4, λ_ex_ = 301 nm, λ_em_ = 397 nm).

To further study the detection ability of Sensor TQA for Fe^3+^, the Stern–Volmer equation was used to estimate the detection limit, where (*I*_0_ − *I*)/*I* is plotted on the *Y*-axis vs. [Fe^3+^] on the *x*-axis, and *I*_0_ is the fluorescence intensity of Sensor TQA, while *I* is the fluorescence intensity of Sensor TQA–Fe^3+^. As illustrated in Figure 5, after linear fitting, the linear equation y = 1257.6675x + 0.00474 (R^2^ = 0.98454, *s* = 0.00706) was obtained. According to the detection limit in Equation (1), *s* is the standard deviation of replication measurements, and *m* is the slope of the calibration curve [22]. The detection limit of Sensor TQA for Fe^3+^ is calculated to be 0.16841 μM.

(1) $$DL=\frac{3s}{m}$$

The fluorescence quantum yields of Sensor TQA and Sensor TQA–Fe^3+^ were determined according to Equation (2), where F_U_ and F_S_ denote the integral fluorescence intensity of the determinand and standard substance; A_U_ and A_S_ denote the relevant maximum absorbance of the determinand and standard substance, respectively (quinine sulphate was used as a reference quantum yield standard, λ_ex_ = 410 nm, quantum yield = 0.54 in 0.1 M H_2_SO_4_). The quantum yields for Sensor TQA and Sensor TQA–Fe^3+^ calculated from the equation in aqueous ethanol were 0.053 and 0.31, respectively.

(2) $$Y_{u}=Y_{S}\times \frac{F_{U}}{F_{S}}\times \frac{A_{S}}{A_{U}}$$

**Figure 6:**
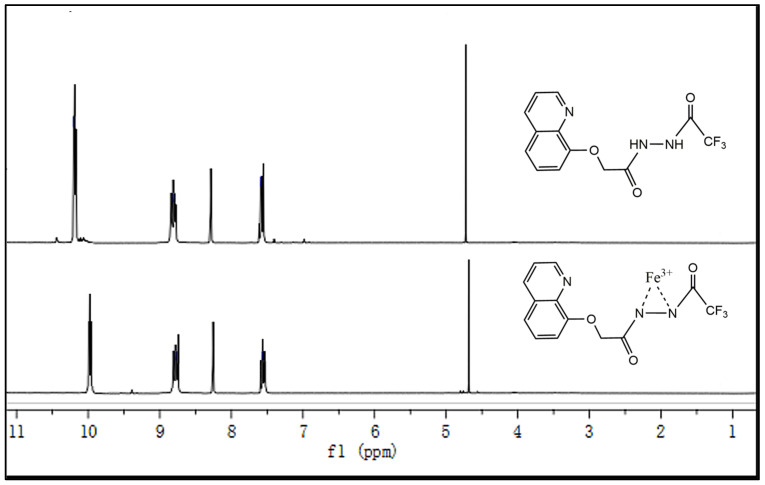
^1^H NMR (300 MHz) spectral changes in Sensor TQA (10 μmol/L) in DMSO-*d*_6_ (containing 10% D_2_O) upon addition of Fe^3+^.

To understand the complexation ability and binding ratio of Sensor TQA molecule with Fe^3+^, the modified Stern–Volmer Equation (3) was used to calculate the complexation constant and complexation ratio of Sensor TQA to Fe^3+^ [23]. In the equation, *K_SV_* is the Stern–Volmer constant; *n* is the number of binding sites; and *Q* is the concentration of Fe^3+^. By taking the logarithm value of the data in Figure 6, the linear equation of the modified Stern–Volmer equation is obtained; it can be calculated that *K_SV_* is 2.767 × 10^3^ M^−1^, and *n* is 1.11. Therefore, the complexation constant of Sensor TQA with Fe^3+^ is 2.767 × 10^3^ M^−1^, and the complexation ratio of Sensor TQA with Fe^3+^ is 1:1.

(3) $$\mathrm{lg}(\frac{I_{0}-I}{I})=\mathrm{lg}\;K_{SV}+\mathrm{nlg}\;Q$$

To obtain further details of the co-ordination between Sensor TQA and Fe^3+^, we carried out ^1^H NMR titration experiments in DMSO-*d*_6_ (containing 10% D_2_O). As shown in Figure 6, upon the addition of Fe^3+^ (10 μmol/L) dissolved in D_2_O/DMSO-*d*_6_ (1:9, *v*:*v*), the chemical shift in the protons of Sensor TQA (10 μmol/L) in DMSO-*d*_6_ (containing 10% D_2_O) changed, especially in the two NH groups, which verified that the two nitrogen atoms are involved in co-ordination with Fe^3+^.

**Figure 7:**
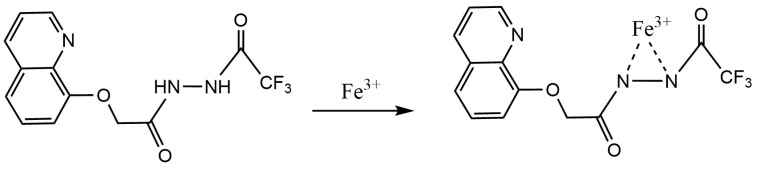
Binding model of Sensor TQA and Fe^3+^.

Based on the experimental results of ^1^H NMR titration and the 1:1 binding ratio between Sensor TQA and Fe^3+^, the possible binding model between Sensor TQA and Fe^3+^ is further speculated, as shown in Figure 7. Based on the electronic configuration of Fe^3+^, the detection mechanism of Sensor TQA may be attributed to the spin forbidden by the n–π transition that occurs when Sensor TQA binds to Fe^3+^ during excitation. The lone-pair electrons of the N atom used for co-ordination undergo non-radiative relaxation, suppressing the original electron transition process and further generating the effect of PET, resulting in significant fluorescence quenching of Sensor TQA.

### 2.2. Theoretical Calculation Research

**Figure 8:**
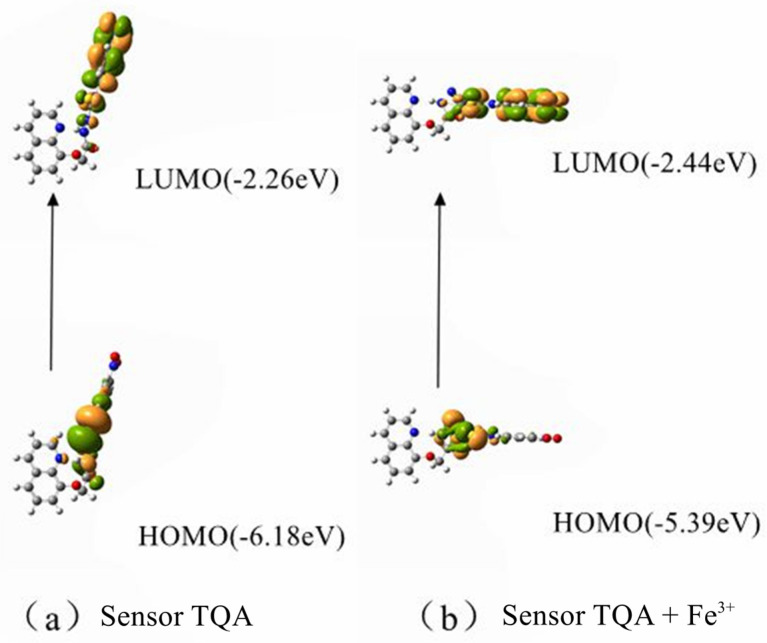
Spatial distribution of HOMO and LUMO of Sensor TQA and Sensor TQA–Fe^3+^.

To better analyze the spatial electron cloud arrangement and sensing mechanism before and after the interaction between Sensor TQA and Fe^3+^, based on quantum chemistry, Gaussian software (Version c01) was used to systematically calculate the spatial electron cloud arrangement of Sensor TQA. Functional theory (PBE0) was selected, and Gaussian 09 software (Version c01) was used for calculations with 3–21G as the basis set. As shown in Figure 8, the HOMO and LUMO of Sensor TQA are mainly distributed on the trifluoroacetyl group, and the arrangement of HOMO and LUMO also indicates that electrons are capable of transitioning well to the excited state. The synthesized Sensor TQA has a good conjugated structure and is rich in electrons, which are able to efficiently bind with Fe^3+^. The emission peak of the fluorescent molecule is generated by S_0_, corresponding to HOMO → LUMO. The fluorescence emission peak of the product molecule is larger than the emission peak wavelength of the sensor molecule, and the large difference in fluorescence signal is conducive to fluorescence detection and practical applications. All theoretical calculation results are in good agreement with actual experimental test data, which also verifies the scientific validity of the experimental results from a theoretical perspective.

**Figure 9:**
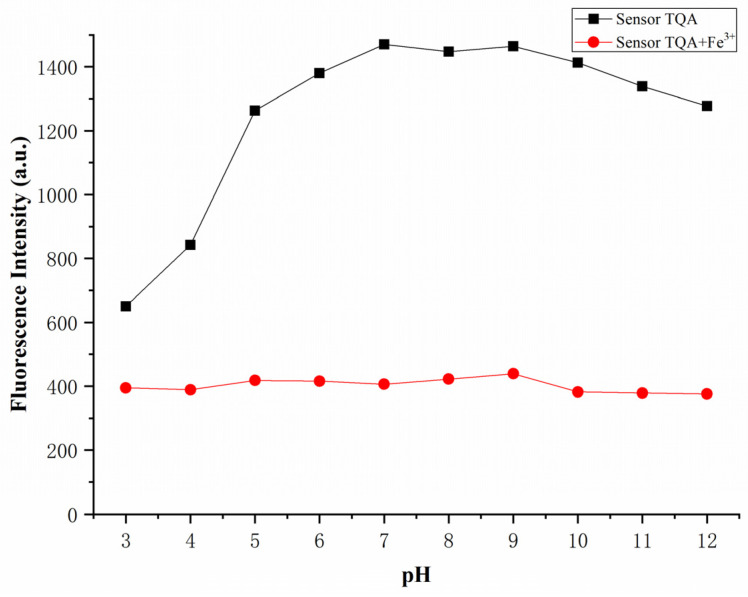
Effect of pH on the fluorescence intensities of Sensor TQA and Sensor TQA–Fe^3+^ in DMF/water solution (*v*:*v* = 1:1, pH = 7.4, λ_ex_ = 301 nm, λ_em_ = 397 nm).

The effects of pH on the fluorescence intensities of Sensor TQA and Sensor TQA–Fe^3+^ were tested to verify their properties under extreme acid-base conditions. In order to maintain the pH under different test conditions, the following buffer systems were added, respectively: HCl (pH = 3–4), NaAc-HAc (pH = 5–6), HEPES-NaOH (pH = 7), NH_3_-NH_4_Cl (pH = 8–11), NaOH (pH = 12). As shown in Figure 9, the independent Sensor TQA could maintain fluorescence emission at pH = 5–12, while the high acidic conditions had an obvious impact on fluorescence intensity. This may be due to the fluorescence quenching caused by high hydrogen ion concentration. Nevertheless, the pH had little effect on Sensor TQA–Fe^3+^; therefore, we believe that Sensor TQA could effectively detect Fe^3+^ within a wide pH range.

### 2.3. Research on Biological Applications of Fluorescence Sensor TQA

**Figure 10:**
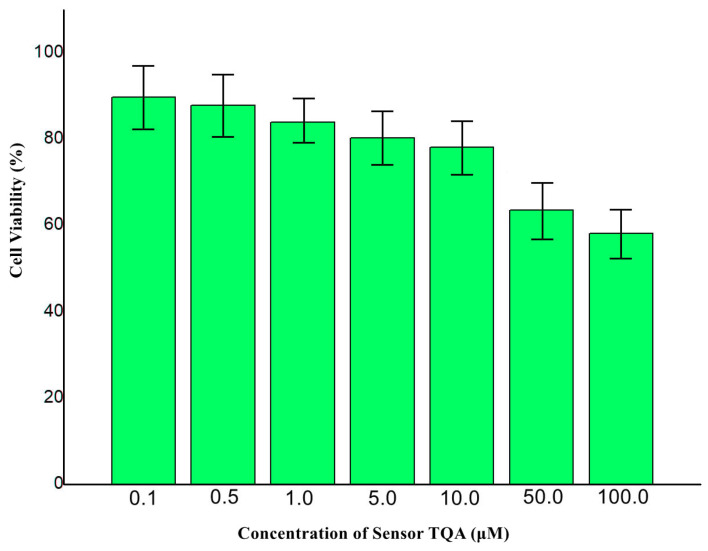
Concentration-dependent cell viability assay for Sensor TQA.

To research the biological application of Sensor TQA, a cytotoxicity test was adopted for live liver cancer cells. A concentration-dependent cell viability assay was conducted under 0.1 μM, 0.5 μM, 1.0 μM, 5.0 μM, 10.0 μM, 100.0 μM Sensor TQA. As illustrated in Figure 10, cell viabilities could maintain a high level under the concentrations of 0.1 µM–10.0 µM. However, when the concentration reached 50 µM, cell viability decreased to below 80%, and the IC_50_ value was ascertained as 201.6 μM, the same level as many reported fluorescent sensors [24,25,26]. Therefore, we believe that the TQA sensor still has certain cytotoxicity. The reason for this may be that the sensor TQA, under high concentration conditions, causes damage to the cell membrane or interferes with cell metabolism, leading to a decrease in cell viability.

**Figure 11:**
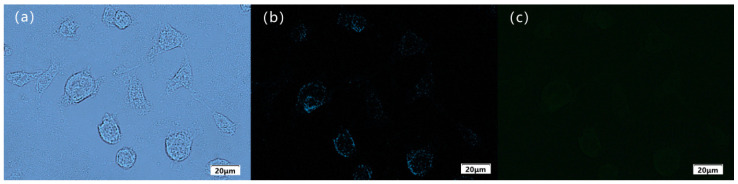
(**a**) Bright-field transmission image of live cells processed with Sensor TQA; (**b**) Fluorescence imaging of live cells processed by Sensor TQA; (**c**) Fluorescence imaging of live cells processed by Sensor TQA–Fe^3+^.

To further investigate the biocompatibility of Sensor TQA and its practical application in the field of biological tracing, live liver cancer cells (HepG2) were selected to further test the Fe^3+^ detection ability of Sensor TQA. First, pre-cultured liver cancer cells were removed from the incubator. A micropipette was used to aspirate the cell culture medium. Cells were rinsed with phosphate-buffered solution (PBS) at pH = 7.4; the process was repeated three times to ensure removal of the culture medium. A volume of 30 µM of Sensor TQA (dissolved in DMSO) was added to the cell culture plate. Cells were cultivated at room temperature for 30 min until Sensor TQA completely entered the liver cancer cells. Rinsing was continued with phosphate-buffered solution (PBS) and repeated three times. Finally, cells were transferred to a fluorescence microscope for observation and imaging. As shown in Figure 11a,b, the contour of the cultured cells is good and exhibits obvious fluorescence inside the cell under a fluorescence microscope, indicating that Sensor TQA is able to penetrate the cell membrane and be absorbed by the cells. Afterward, the prepared Fe^3+^ (30 µM) solution was further added to the cells and cultured for 30 min for fluorescence imaging. As shown in Figure 11c, the fluorescence intensity of the cells significantly decreased, with almost no fluorescence. In the fluorescence imaging experiment, it can be found that Sensor TQA is able to penetrate the cell membrane and enter the cell to recognize Fe^3+^, indicating that Sensor TQA has good biocompatibility and potential applications in the field of biological tracing.

**Figure 12:**
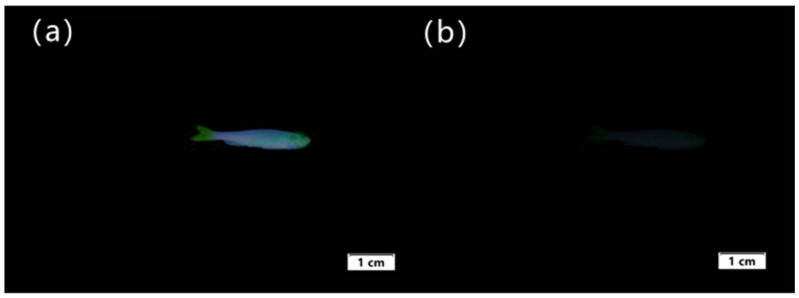
Image of adult zebrafish under UV light at 365 nm: (**a**) Zebrafish cultivated with Sensor TQA; (**b**) Zebrafish cultivated with Sensor TQA and Fe^3+^.

After verifying that Sensor TQA is capable of performing fluorescence imaging in cells, we continued to study its biosensing performance and selected zebrafish for the animal specimen experiments. Zebrafish is a common tropical fish with strong vitality and a similarity of up to 87% with human genes. It is approximately 2–4 cm in length and is usually used for biological imaging experiments. Therefore, we further validated the biocompatibility of Sensor TQA using zebrafish. At room temperature, zebrafish were cultured in sterile water for 24 h. After changing the water three times, the cultured zebrafish were added to an aqueous solution containing Sensor TQA (30 µM) and cultured for 1 h. The zebrafish were then removed and washed three times with HEPES-NaOH buffer solution. Subsequently, fluorescence imaging experiments were conducted. In Figure 12a, it can be found that zebrafish absorbed Sensor TQA and exhibited significant fluorescence emission. The morphology of zebrafish can be clearly observed. Afterward, Fe^3+^ was added to the culture medium to further culture the zebrafish for 1 h. After washing the zebrafish three times with the HEPES-NaOH buffer solution, the image of zebrafish under UV light shown in Figure 12b was obtained. It is difficult to observe fluorescence emission from zebrafish, and only the outline of zebrafish is roughly observed. The experimental results showed that Fe^3+^ added to the culture medium reacted chemically with Sensor TQA-covered zebrafish, resulting in a significant decrease in fluorescence intensity. This indirectly proves the biocompatibility of Sensor TQA, which can be used in the field of practical biological imaging and has certain application value.

## 3. Materials and Methods

### 3.1. Materials

The chemicals and reagents involved in this research were purchased from commercial suppliers (Aladdin Reagent, Shanghai, China) and used without further purification. The solvents for spectra detection were HPLC reagents without fluorescent impurity. Solutions of different ions (Cu^2+^, Ca^2+^, Ba^2+^, Hg^2+^, Fe^3+^, Mn^2+^, Na^+^, Mg^2+^, Co^2+^, Cd^2+^, Zn^2+^, Pb^2+^) in titration experiments were made from CuCl_2_, CrCl_3_·6H_2_O, CaCl_2_, FeCl_2_, Hg(NO_3_)_2_, FeCl_3_, Mn(NO_3_)_2_·6H_2_O, NaNO_3_, MgCl_2_, CoCl_2_, CdCl_2_, ZnCl_2_, Pb(NO_3_)_2_ and dissolved in HEPES-NaOH buffer solution at pH = 7.4. The anions (NO_3_^−^, F^−^, Cl^−^, Br^−^, I^−^, Ac^−^, SO_4_^2^^−^) used in the experiment on sensor response to anions were made from NaNO_3_, C_4_H_12_FN, C_4_H_12_ClN, C_4_H_12_BrN, C_4_H_12_IN, CH_3_COONa, Na_2_SO_4_ and dissolved in HEPES-NaOH buffer solution at pH = 7.4.

### 3.2. Methods

Experimental Intermediate and Sensor TQA were characterized by ^1^H NMR, ^13^C NMR (Varian mercury-300 spectrometer, Palo Alto, CA, USA) and HRMS analyses [Agilent 1290-micro TOF QII (Santa Clara, CA, USA), LC column: CenturySILC18-AQ + 5 mm (50 × 4.6 mm, 5 micron); mobile phase: the mobile phase consisted of acetonitrile–water (90:10, *v*/*v*); auto-sampler: reconstituted samples were loaded in the auto-sampler, and the injection volume was 40 mL of the sample extract, test conditions: 25 °C]. Relevant data are provided in the supporting information (Figures S1–S7). UV–vis spectra were obtained with an Shimadzu UV-2600 (Kyoto, Japan), using Plastibrand PMMA cuvette (Labshark, Changde, China) (d = 1 cm) in the wavelength range of 280–500 nm. The fluorescence spectra were obtained with a Hitachi F-4500 spectrofluorimeter (Tokyo, Japan), using quartz cell (d = 1 cm) in the wavelength range of 300–500 nm; the excitation and emission slits were both 5 nm. The test instrument for the pH was a Mettler–Toledo Instrument DELTE 320 pH (Parramatta, NSW, USA). The fluorescent cell image experimental device consisted of an Olympus IX-70 fluorescence microscope and an Olympus c-5050 digital camera (Tokyo, Japan).

An MTT assay was used to compare the cytotoxicity of Sensor TQA in different concentrations on live liver cancer cells and determine the dose–effect relationship. Live liver cancer cell density was 4 × 10^5^ per mL, and the cells were seeded on 96-well plates, 100 μL per well. After 24 h of incubation, different concentrations of test substances (dissolved in DMSO) were added. After 48 h of incubation, 20 μL of the MTT (5 mg/mL) solution was added to each well. After 4 h of incubation, 100 μL of 10% SDS solution was added. After 16 h, absorbance at 570 nm was recorded, and the logarithms of the dose and cell inhibition rates were fitted using software (Origin 2018), and the IC_50_ value was calculated [27].

### 3.3. Synthesis

**Scheme 1:**
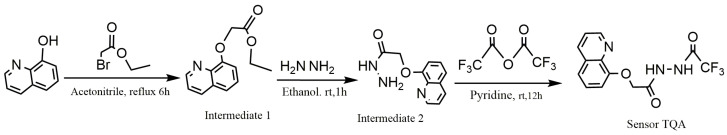
Synthesis of Sensor TQA.

Anhydrous potassium carbonate (3.8 g, 28 mmol) and 8-hydroxyquinoline (2.0 g, 14 mmol) are dissolved in 50 mL acetone and stirred continuously at room temperature for 30 min; then, ethyl bromoacetate is added (2.5 g, 15 mmol) at room temperature and stirred constantly for 6 h. The product is extracted with deionized water and dichloromethane three times. The organic phase is dried with anhydrous magnesium sulfate for 24 h; then, the solvent is evaporated to obtain the crude product. The crude product is purified with column chromatography (developing agent: petroleum ether/ethyl acetate = 2/1) to obtain Intermediate 1 as reddish brown oil (0.9 g). Intermediate 1 (0.50 g, 2.2 mmol) is dissolved in 5 mL methanol; then, 5 mL of the hydrated hydrazine methanol solution (2.0 mol/L) is added dropwise to the Intermediate 1 solution; then, constant stirring is applied at room temperature for 1 h, and the resulting white precipitate is collected and purified with column chromatography (developing agent: ethanol/dichloromethane = 1/1) to obtain Intermediate 2 (0.39 g) as white solid. The yield is 69%. ^1^H NMR (300 MHz DMSO-*d*_6_, 25 °C, 10 μmol/L, TMS) δ: 9.46 (s, 1H), 8.91 (d, 1H, *J* = 3.0 Hz), 8.36 (d, 1H, *J* = 6.0 Hz), 7.56 (m, 3H, *J* = 3.0 Hz), 7.27 (d, 1H, *J* = 6.0 Hz), 4.76 (s, 2H), 4.40 (s, 2H). ^13^C NMR (75 MHz DMSO-*d*_6_, 25 °C, 10 μmol/L, TMS) δ: 167.32, 154.42, 149.88, 140.33, 136.51, 129.6, 127.23, 122.48, 121.42, 112.19, 68.77. ESI-MS *m*/*z* [M]^+^ calc. 217.09, fobs. 218.1 (Figures S1–S3). Intermediate 2 (0.3 g) and excess trifluoroacetic anhydride (0.87 g) are dissolved in 30 mL pyridine; stirring is applied continuously at room temperature for 12 h; then, the solvent is evaporated to obtain the crude product. The crude product is purified with column chromatography (developing agent: petroleum ether/ethyl acetate = 2/1) to obtain the end product 2,2,2-trifluoro-*N*′-(2-(quinolin-8-yloxy)acetyl)acetohydrazide (TQA) 0.34 g. The yield is 80%. The synthesis route is shown in Scheme 1. ^1^H NMR (300 MHz DMSO-*d*_6_, 25 °C, 10 μmol/L, TMS) δ: 10.20–10.18 (m, 2H, *J* = 3.0 Hz), 8.90–8.84 (m, 3H, *J* = 3.0 Hz), 8.35 (s, 1H), 7.66–7.62 (m, 2H, *J* = 3.0 Hz), 4.77 (s, 2H). ^13^C NMR (75 MHz DMSO-*d*_6_, 25 °C, 10 μmol/L, TMS) δ: 172.13, 167.32, 154.42, 149.83, 140.33, 136.51, 129.00, 127.23, 122.48, 121.42, 118.19, 112.39, 68.77. ESI-MS *m*/*z* [M]^+^ calc. 313.07, obs. 313.3 (Figures S4–S6).

## 4. Conclusions

In this study, a new Fe^3+^ fluorescence sensor was successfully designed and synthesized by selecting quinoline derivatives as fluorescent groups. The synthesis route of Sensor TQA is simple, and it has good selectivity and sensitivity to Fe^3+^. It is able to overcome external interference and achieve efficient recognition of Fe^3+^. The calculated fitting results indicate that Sensor TQA has a detection limit of 0.16841 μM for Fe^3+^ in a 1:1 binding mode; the complexation constant is calculated to be 2.767 × 10^3^ M^−1^. The quantum chemistry theoretical calculation results for Sensor TQA are highly consistent with the actual experimental results, which also theoretically explains the mechanism of interaction between Sensor TQA and Fe^3+^. At the same time, Sensor TQA was successfully applied in live cell fluorescence imaging and biological imaging. Compared with the other reported quinoline-based sensors for Fe(III), Sensor TQA not only shows good selectivity and sensitivity but also adopts NMR titration, the Stern–Volmer equation and theoretical calculation to explore the co-ordination mechanism in detail. In addition, the good biocompatibility also proves its potential application in the field of biological tracing.

## Data Availability

The data presented in this study are available in Supplementary Material.

## Supplementary Materials

The following supporting information can be downloaded at: https://www.mdpi.com/article/10.3390/molecules30071579/s1, Figure S1. ^1^H NMR (300 MHz) spectrum of intermediate 2; Figure S2. ^13^C NMR (75 MHz) spectrum of intermediate 2; Figure S3. LC-MS of intermediate 2; Figure S4: ^1^H NMR (300 MHz) spectrum of Sensor TQA; Figure S5: ^13^C NMR (75 MHz) spectrum of Sensor TQA; Figure S6: LC-MS of Sensor TQA; Figure S7. Fluorescence response of common anions to Sensor TQA in DMF/water solution.

## References

[1] Theil E., Goss D. (2009). Living with iron (and oxygen): Questions and answers about iron homeostasis. Chem. Rev..

[2] Que E., Domaille D., Chang C. (2008). Metals in neurobiology: Probing their chemistry and biology with molecular imaging. Chem. Rev..

[3] Sahoo S., Sharma D., Bera R., Crisponic G., Callan J. (2012). Iron(III) selective molecular and supramolecular fluorescent probes. Chem. Soc. Rev..

[4] Barb W., Baxendale J., George P., Hargrave K. (1951). Reactions of ferrous and ferric ions with hydrogen peroxide. Part II.—The ferric ion reaction. Trans. Faraday Soc..

[5] Baldwin G. (2009). Gastrins, iron and colorectal cancer. Metallomics.

[6] Wang M., Wang G.X., Xiao F.N., Zhao Y., Wang K., Xia X.H. (2013). Sensitive label-free monitoring of protein kinase activity and inhibition using ferric ions coordinated to phosphorylated sites as electrocatalysts. Chem. Commun..

[7] Yin H.Y., Xu L.B., Porter N. (2011). Free radical lipid peroxidation: Mechanisms and analysis. Chem. Rev..

[8] Kowalczyk M., Mergo P. (2024). Doped epoxy resins as an alternative to luminescent optical sensors. Appl. Sci..

[9] Koralli P., Mouzakis D.E. (2021). Advances in wearable chemosensors. Chemosensors.

[10] Kollur S.P., Shivamallu C., Prasad S.K., Veerapur R., Patil S.S., Cull C., Coetzee J., Amachawadi R. (2021). Recent advances on the development of chemosensors for the detection of mercury toxicity: A review. Separations.

[11] Kolcu F., Erdener D., Kaya I. (2020). A Schiff base based on triphenylamine and thiophene moieties as a fluorescent sensor for Cr (III) ions: Synthesis, characterization and fluorescent applications. Inorg. Chim. Acta.

[12] Yan Y.H., Yang H.Z., Liu H.Z. (2020). Silsesquioxane-based fluorescent nanoporous polymer derived from a novel AIE chromophore for concurrent detection and adsorption of Ru^3+^. Sens. Actuators B-Chem..

[13] Wu M.X., Yang Y.W. (2019). A fluorescent pillarene coordination polymer. Polym. Chem..

[14] Gao N., Yu J., Tian Q., Shi J., Zang L. (2021). Application of PEDOT: PSS and its composites in electrochemical and electronic chemosensors. Chemosensors.

[15] Kalecki J., Iskierko Z., Cieplak M., Sharma P.S. (2020). Oriented immobilization of protein templates: A new trend in surface imprinting. ACS Sensors.

[16] Tigreros A., Portilla J. (2020). Recent progress in chemosensors based on pyrazole derivatives. RSC Adv..

[17] Chatterjee A., Pan N., Maji T., Pasha S., Singh S., Ahmed S., Al-Thakafy J., Pal S. (2021). Highly sensitive optical sensor for selective detection of fluoride level in drinking water: Methodology to fabrication of prototype device. ACS Sustain. Chem. Eng..

[18] Tanwar A., Parui R., Garai R., Chanu M., Iyer P. (2022). Dual “static and dynamic” fluorescence quenching mechanisms based detection of tnt via a cationic conjugated polymer. ACS Meas. Sci. Au..

[19] Mizuno H., Fukuhara G. (2020). Hydrostatic-pressure spectroscopy: Application to functional molecules and supramolecular assemblies. Bunseki Kagaku..

[20] Rios M.C., Bravo N.F., Sanchez C.C., Portilla J. (2021). Chemosensors based on N-heterocyclic dyes: Advances in sensing highly toxic ions such as CN^−^ and Hg^2+^. RSC Adv..

[21] Dumbare S., Doshi A., Ravindran S. (2021). Rhodamine B and rhodamine 6g based sensing of copper ions in environmental and biological samples: Recent progress. Pol. J. Environ. Stud..

[22] Sun M.D., Guo J., Yang Q.B., Xiao N., Li Y.X. (2014). A new fluorescent and colorimetric sensor for hydrazine and its application in biological systems. J. Mater. Chem. B.

[23] Li Y.L., Zhou C., Li J.X., Sun J. (2024). A new phenothiazine-based fluorescent sensor for detection of cyanide. Biosensors.

[24] Xie D., Jing J., Cai Y., Tang J., Chen J., Zhang J. (2014). Construction of an orthogonal ZnSalen/Salophen library as a colour palette for one- and two-photon live cell imaging. Chem. Sci..

[25] Li T., Xiao X., Zhou C., Luo M. (2024). Design and application of Cu^2+^ fluorescent sensor based on carbazole derivatives. J. Fluoresc..

[26] Li J., Zhou C., Zhang H., Hou Y., Pan Q., Sun J., Li X. (2022). A novel colorimetric and ‘‘turn-on” fluorescent sensor for selective detection of Cu^2+^. Arab. J Chem..

[27] Benov L. (2021). Improved Formazan Dissolution for Bacterial MTT Assay. Microbiol. Spectr..

