# Supplementary material for: A Novel Fluorescent Sensor for Fe3+ Based on a Quinoline Derivative
Source: Molecules. 2025 Apr 1;30(7):1579. doi: 10.3390/molecules30071579 (PMC11990252; doi:10.3390/molecules30071579)
Supplement: Supplementary file 1 [file molecules-30-01579-s001.zip › molecules-3500116-supplementary.pdf]

A Novel Fluorescent Sensor for Fe^{3+} Based on a Quinoline Derivative

Xinru Zhang [1], Bo Sun [2], Huan Zhang [1], Chen Zhou [1,*], Qingqing Pan [1,*], Yining Wang [1], Chenyang Zou [1], Juan Hou [1] and Jing Sun [1]

[1] School of Chemistry & Environmental Engineering, Jilin Provincial International Joint Research Center of Photo-Functional Materials and Chemistry, Changchun University of Science and Technology, Changchun 130022, China; 18265510750@163.com (X.Z.); devilzc@163.com (H.Z.); yiningwang@cust.edu.cn (Y.W.); 2021800026@cust.edu.cn (C.Z.); houjuan0503@126.com (J.H.); sunjing@cust.edu.cn (J.S.)

[2] China National Petroleum Corporation Hohhot Petrochemical Branch, Hohhot 010010, China; jh_sunb01@petrochina.com.cn

* Correspondence: zhouchen@cust.edu.cn (C.Z.); panqq349@nenu.edu.cn (Q.P.)

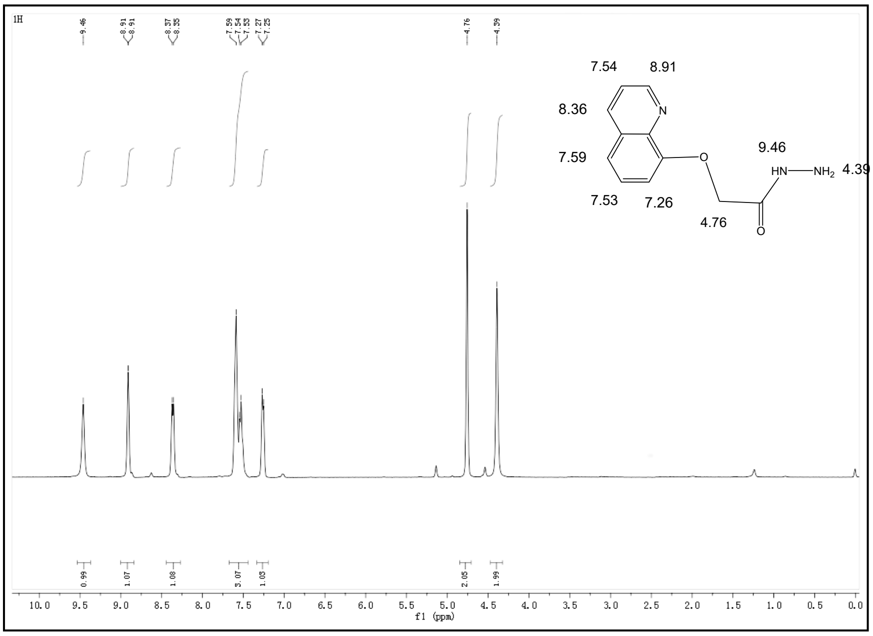

Figure S1. ^1H NMR (300 MHz) spectrum of intermediate 2 (10μmol/L) in DMSO-d_6

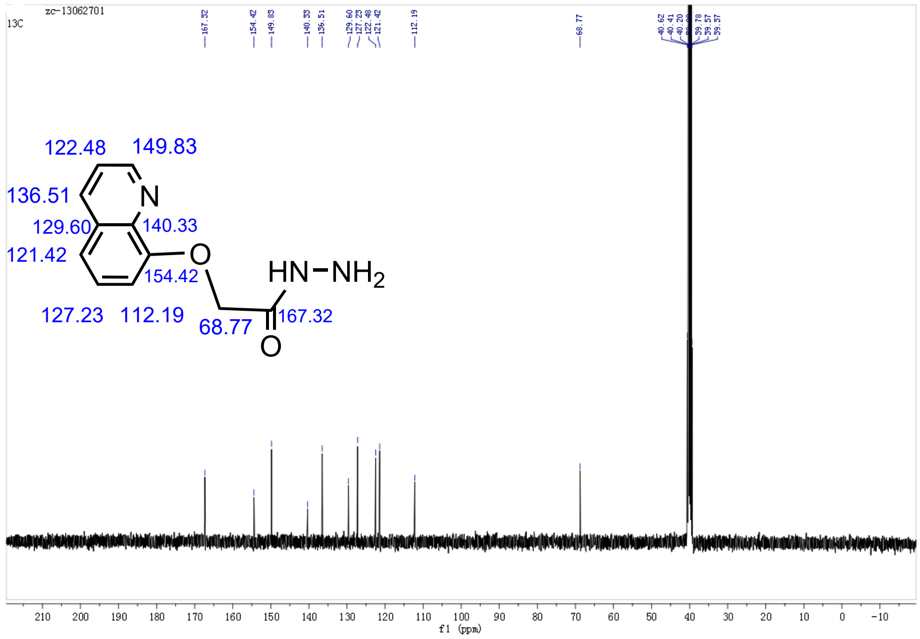

Figure S2. ^{13}C NMR (75 MHz) spectrum of intermediate 2 (10μmol/L) in DMSO-d_6

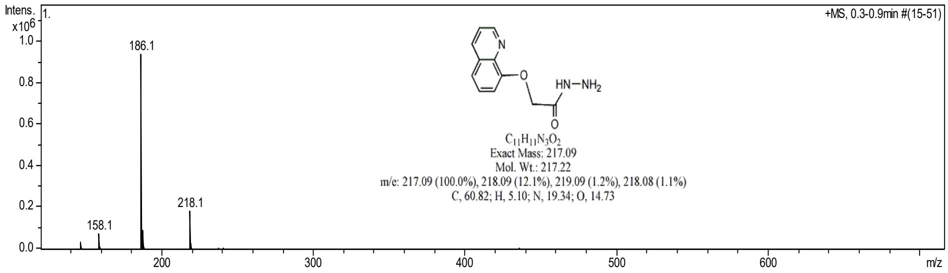

Figure S3. LC-MS of intermediate 2

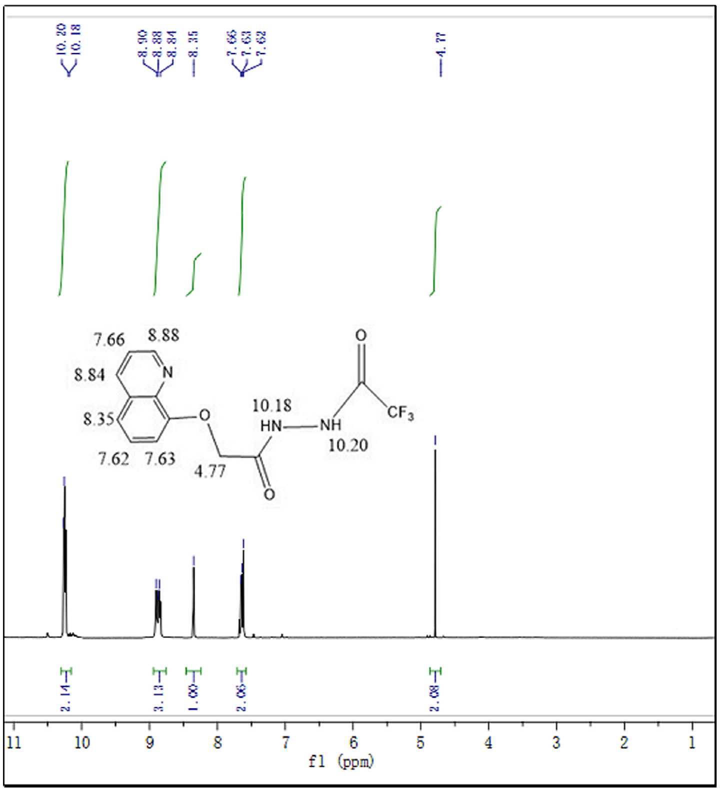

Figure S4. ^1H NMR (300 MHz) spectrum of Sensor TQA (10µmol/L) in DMSO-d_6

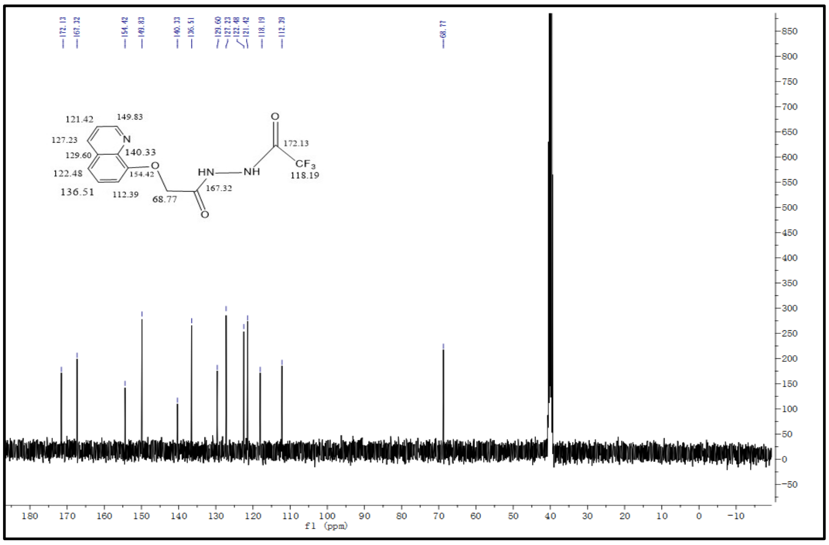

Figure S5. ^{13}C NMR (75 MHz) spectrum of Sensor TQA (10µmol/L) in DMSO-d_6

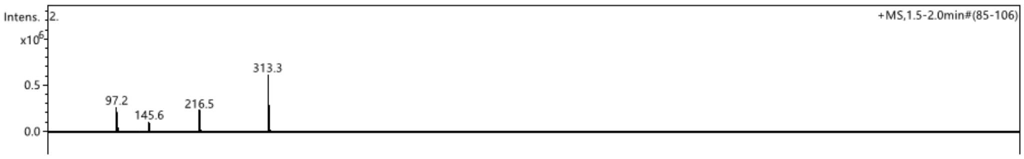

Figure S6. LC-MS of Sensor TQA

Chemical Formula: $C_{13}H_{10}F_3N_3O_3$

Exact Mass: 313.07

Molecular Weight: 313.24

m/z: 313.07 (100.0%), 314.07 (14.3%), 315.07 (1.7%), 314.06 (1.1%)

Elemental Analysis: C, 49.85; H, 3.22; F, 18.20; N, 13.42; O, 15.32

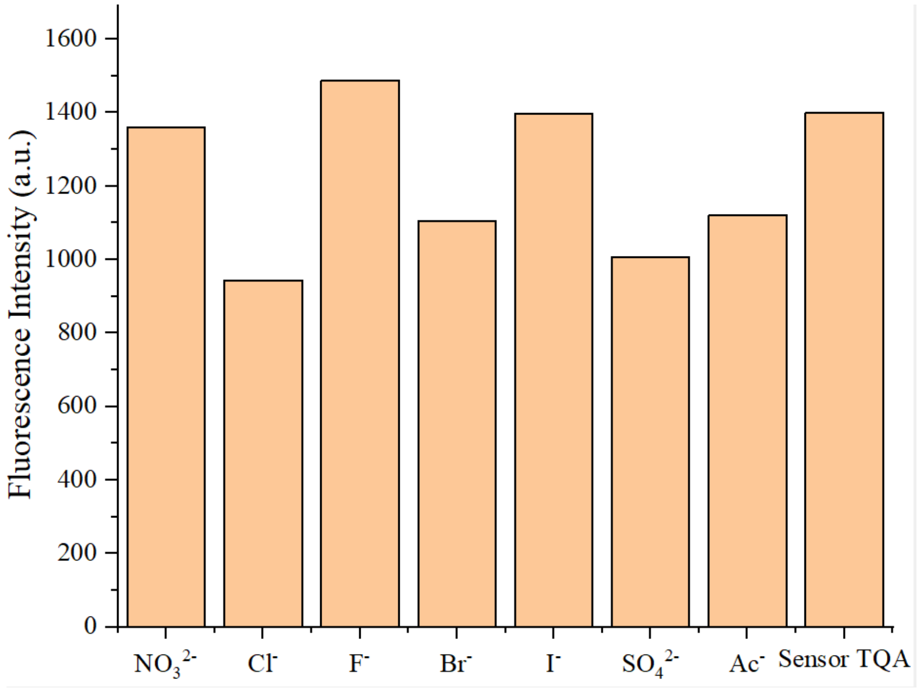

Figure S7. Fluorescence response of common anions to Sensor TQA in DMF/water solution (v:v=1:1, pH 7.4, λ_{ex}=301 nm, λ_{em}=397 nm)
